# Transitioning from a Multi-Agency to an Integrated Food Control System: A Case Study from the Sultanate of Oman

**DOI:** 10.3390/foods14152618

**Published:** 2025-07-26

**Authors:** Moza Abdullah Al Busaidi, Mohammad Shafiur Rahman, Hussein Samh Al Masroori

**Affiliations:** 1 Ministry of Higher Education, Research and Innovation, (Former), P.O. Box 92, Muscat 123, Oman; 2 Oman Consumer Protection Association, P.O. Box 1691, Ruwi, Muscat 112, Oman; 3 Department of Food Science and Nutrition, College of Agricultural and Marine Sciences, Sultan Qaboos University, P.O. Box 34, Muscat 123, Oman; shafiur@squ.edu.om; 4 Food Safety and Quality Center, Ministry of Agriculture, Fisheries and Water Resources, P.O. Box 438, Muscat 100, Oman; masroori@squ.edu.om; 5 Department of Marine Science and Fisheries, College of Agricultural and Marine Sciences, Sultan Qaboos University, P.O. Box 34, Muscat 123, Oman

**Keywords:** food safety management, integrated food control system, food security, multi-agency approach, legislative framework, comparative analysis

## Abstract

Food safety regulations and their implementations are becoming increasingly complex due to various reasons such as diverse food sources, supply chain, processing technologies, distribution systems and environmental concerns. Additionally, it is crucial to address diversified consumers and their preferences. To address these multifaceted challenges, adopting an integrated unified management system is essential. This review provides a comprehensive overview of the progressive food safety governance in the Sultanate of Oman. The country is transitioning from a multi-agency to an integrated food control management system. This integrated approach can enhance the coordination between different government agencies and other stakeholders, avoid duplication, identify required resources and ensure optimum use of the resources. The progress can enhance efficiency and effectiveness in managing food safety in Oman. It addresses the issues of the food safety management system, explores the legislative frameworks, risk-based assessment and their enforcement, and creates public awareness and required research for continuous improvement in food safety. This integration approach is expected to continue strengthening food safety governance in the country. Finally, future challenges in achieving food safety are envisioned, including new food sources and technologies, applications of artificial intelligence, and new sensors for quick identification of risks in foods.

## 1. Introduction

Effective national food control systems (NFCSs) are crucial in safeguarding consumer health and well-being [1]. The NFCSs are essential structures designed to protect all the processes of the food supply chain consisting of the initial production stages to the final consumer. They play a vital role in ensuring safety and quality of food products throughout its lifecycle. The effectiveness of NFCSs often depends on its human and capital capacity, public health status and economic stability. These factors help to build trust and confidence both domestically and in international trade performance [2]. Active collaboration and engagement from all stakeholders in the food supply chain are essential for adopting a preventive approach and developing a comprehensive strategy that spans the entire food chain. This approach can enhance national food security and build integrity and credibility in the global marketplace [3]. Furthermore, NFCSs contribute to the achievement of the UN Sustainable Development Goals SDG 2 (Zero Hunger) and SDG 3 (Good Health and Well-Being) by safeguarding national health and promoting fair practices in the food trade [4].

Establishing effective and modern food safety systems depend on several factors, including expertise in the field, sound policies, a strong food safety culture, adequate resources, adherence to food laws, and compliance with standards and regulations [1]. The foundation of a resilient national food control system lies in the formulation and enforcement of comprehensive food laws and regulations, which ensure the safety and quality of food products [3,5]. Ensuring food safety and quality is of paramount importance for the protection of public health, promoting consumer confidence and enhancing food security. Effective management of food safety and quality throughout the farm-to-plate continuum improves the availability of wholesome food, protects consumers from mislabeled or adulterated food products, and reduces the incidence of foodborne illnesses, which affect millions of lives worldwide causing numerous fatalities annually [5,6].

With escalating demand, complex supply chains, and increased global interdependence, ensuring food safety has become a critical challenge. Having consolidated food control systems that are more unified with streamlined functions, mandates and a risk-based approach offers numerous benefits, thus enhancing efficiency [1,3,7]. National food control systems around the globe have transitioned from a multi-agency structure to integrated or more unified (single) food control systems [8,9,10]. These unified systems have shown a reduction in fragmentation, ensuring a quicker and consistent response to food safety challenges, elimination of redundancies, consistency in regulations and standards, efficient management, cost reduction of monitoring and surveillance and resource optimization. They are more aligned with international food safety standards and global trade with better surveillance and data sharing. Many countries have shown successful transition and overcome institutional resistance, fragmentation, and the complexity of integrating multiple agencies and functions.

Oman made the transition from the multi-agency structure to an integrated system in 2020 with the issuance of Royal Decree No. (92/2020) by appointing the Ministry of Agricultural, Fishery, and Water Resources as the competent authority for food safety in Oman [11,12]. This transition has involved major legal reforms, capacity building and engagement of various stakeholders to create a cohesive framework by strengthening the country’s food safety and regulatory capabilities.

### 1.1. Food Security and Sustainability in Oman

According to the Global Food Security Index, food security is described as the condition where individuals consistently have physical, social, and economic access to an adequate and nutritious food supply to maintain a healthy and active lifestyle [13]. It consists of four main pillars for maintaining food supply: food availability, food access, food utilization and food stability [14]. Food safety and food security are interconnected elements essential for a sustainable future and should be aligned to achieve the UNSDGs, and innovative strategies, such as reducing food waste, promoting plant-based diets, and valorization of food resources from conventional inedible parts and byproducts [15].

Due to its historically low food production levels, Oman has made significant efforts to achieve self-sufficiency in food security across various sectors, including agriculture and fisheries. These efforts have been driven by strategic planning and the adoption of modern technologies. By 2040, the country aims to reach full food sufficiency [16]. This progress aligns with the aims of setting out in the tenth Five-Year Economic Development Plan (2021–2025) and the Oman Vision 2040 [17].

Significant achievements have been observed with a substantial rise in food production, from 3.9 million tons in 2019 to 4.7 million tons in 2022, reflecting an average annual growth rate of 6.4% and with a value increase from RO958 million to RO1.261 billion for 2019 and 2022, respectively, marking a 9.6% increase [18]. Similarly, the country has made notable advances in self-sufficiency across various food sectors. In 2022, it reached 97% self-sufficiency in dates, 88% in fresh milk, 77% in vegetables, 61% in poultry, 59% in table eggs, 44% in red meat, 26% in fruits and recording the highest self-sufficiency in fish products at 151%. The agriculture and fisheries sectors contributed 2.1% to the country’s GDP, with an increase of 3.5% in their share of non-oil GDP [18].

In order to enhance food security and to align with the tenth Five-Year Economic Development Plan (2021–2025) and Oman Vision 2040, the Ministry of Agriculture, Fisheries and Water Resources (MAFWR) launched an investment program known as the “Food Security Laboratory”. It is a collaboration effort between the MAFWR, the Oman Vision 2040 Follow-up and Implementation Unit, the National Investment and Export Development Program (Nazdaher), and the National Employment Program [19]. The event has been held annually since 2021, aiming to empower the country’s food security by introducing new investment opportunities to boost the food sector’s contribution to GDP and enhancing food sustainability and availability.

In the 11th edition of the Global Food Security Index, 113 countries were evaluated based on key food security dimensions, such as food accessibility, availability, quality and safety, sustainability, and adaptation [13]. Oman was ranked 35th globally in 2022 with an improved score from 40th place in 2021. It held third in the Arab world with a score of 71.2 points after the United Arab Emirates ranked first in the Arab world (23rd globally), followed by Qatar in second place (30th globally) [13]. Oman performed well in several key indicators within the Arab World, ranking second for food accessibility, with a score of 88.6 points, and showed notable improvement in food availability, rising to fourth place with an increase of 7 points in its score compare to eighth back in 2021. Furthermore, it gained seventh position in the Arab world within the food sustainability and adaptation category, improving of 8.4 points.

In the Quality and Safety Index, Oman ranked 42nd globally with a score of 73.2% [13]. Within the Arab region, Oman maintained its position as the third highest ranked country in the food safety and quality index, consistent with its ranking in 2021. However, the country showed a declining score, from 83.8 points to 73.2 points, in the years 2021 and 2022, respectively [20]. One main reason for the decline was due to the low score received in the legislation component. This component reflects the progress, update and amendment of local legislations in the last 5 years. It stated that there were no changes in the regulation, while in reality there were a few additions and amendments in Oman’s food safety regulation, which were not reflected in the assessment.

### 1.2. Regional and Global Interaction and Collaboration

Oman has been actively participating in various regional and international food safety organizations, in particular the Gulf Cooperation Council (GCC). Being a member of this cooperation with other member states including the Kingdom of Saudi Arabia, the State of Qatar, the United Arab Emirates, the Kingdom of Bahrain, and the State of Kuwait, the council has been instrumental in eliminating trade barriers and felicitating trade practices among its members [21]. Harmonization of standards and technical regulations by the Gulf Standards Organization (GSO) has eased the challenges faced by policy makers, food traders and producers [22]. A unified GCC Custom Union with the aim of creating a free trade zone, a single entry and a common market for the GCC member states to examine and ensure conformity of any foreign goods. Thereafter, goods can move freely within member states overcoming the intra-GCC transit of goods given that member states operate as a single customs territory [21].

Many efforts have been made within the GCC member states to enhance food safety requirements, with mutual coordination to mitigate risks associated with the dependency of imported goods [23,24]. Advancement in the development of the food safety legal frameworks has made significant strides within member states. A unified food law is currently under discussion by the Ministerial Committee for Food Safety of the Gulf Cooperation Council (GCC) to fully or partially replace the current national law of each country. The decision aims to enhance enforcement and monitoring practices in achieving integration among the member states in safeguarding their public health. Furthermore, a GCC Guide for Control on Imported Foods has been developed by the Secretariat General of the Cooperation Council for the Arab States of the Gulf and adopted by the GCC member states [24]. The guide aims to achieve unified and harmonized inspection systems and certifications for imported goods based on risk management, best practices and in alignment with World Trade Organization agreements, Codex Alimentarius, World Organization for Animal Health (WOAH) and the International Plant Protection Convention (IPPC) [24]. Consequently, imported food entering any of GCC entry points should undergo health control checks, and carry verified health certificates. A GCC Rapid Alert System for Food and Feed (GRASFF), established in 2012, represents a significant advancement for the Gulf Cooperation Council in the food safety domain [23,24,25]. The system enables rapid exchange of information during food related emergencies or crises and is regarded as one of the most comprehensive risk-based systems implemented in the region.

Oman has been actively engaged on the global stage as a member of several key organizations, including Codex Alimentarius, World Trade Organization, World Health Organization (WHO) and the Food and Agriculture Organization (FAO). During the 47th Session of the Codex Alimentarius Commission (CAC47), Oman took the role of Coordinator of the FAO/WHO Codex Coordinating Committee for the Near East. This international involvement has played a significant role in strengthening the country’s food safety framework by aligning food safety practices with global benchmarks, benefiting from knowledge exchange, and adopting best practices in food safety management.

In addition to Oman’s perspective, international initiatives would be interesting to discuss. Martin et al. [26] outlined the progress of the food safety regulations and their implementation in Australia. Progress was driven to reduce inconsistencies and inefficiencies with State and Territory legislation, and to reduce the cost and link with the international regulatory system. The States and Territories are now implementing this system. Similarly, Slorach [27] emphasized the integrated approaches to the management of food safety throughout the food chain. The regulatory governance pathways to improve the efficacy of Australian food policies was recently published considering a specific case [28].

## 2. Objectives

This review provides a comprehensive overview of the recently restructured food safety governance in the Sultanate of Oman, highlighting the process of transitioning from a multi-agency to an integrated food control system. The primary objective is to assess the transition that has affected the coordination, efficiency and the overall effectiveness in managing food safety in the country. To achieve this, the manuscript examines key components of the national food safety system, including legislative and regulatory frameworks, risk assessment and enforcement mechanisms, public awareness and the role of research and development. The review also explores how the recent restructuring has improved food safety governance in Oman, the major achievements and challenges encountered during this transition and identifying areas within the current framework that require further enhancement to support continuous improvement. By addressing these questions, the study provides a comprehensive understanding of the current dynamics of food safety governance in the Sultanate of Oman and provides insights to support future policy and institutional development.

## 3. Review Methodology

The study employed a qualitative approach guided by key frameworks and guidelines developed by the FAO and WHO [1,3,6,7]. The methodology focused on examining the restructuring of food safety governance in the Sultanate of Oman in accordance with Royal Decree No. (92/2020).

The analytical work was guided by the following components:

• The five main core elements of national food safety system by FAO/WHO [3,7] in terms of
  ○ Food control management;
  ○ Food legislation;
  ○ Food inspection and surveillance;
  ○ Official food control laboratories;
  ○ Food safety and quality information, education and communication.
• The FAO/WHO Food Control System Assessment tool [1] was incorporated into the study to enhance the assessment, and to evaluate the newly integrated system in the country based on four dimensions, including inputs and resources, control functions, stakeholder interaction, science and knowledge, and continuous improvements.
• The FAO/WHO Guide to Developing National Food Recall Systems [6] was used to assess Oman’s emergency response and food recall capabilities.

A structured search approach was also employed to identify relevant sources, and involved three main source types:

• Official policy documents, legislation, government databases and reports. The search method included royal decrees, ministerial decisions, national strategy plans and internal regulatory reports and documents. These documents were collected through governmental portals, websites, official gazettes and legal databases.
• Stakeholder semi-structured interviews: the interviews were carried out with key officials from various governmental authorities.
• Peer-reviewed literature, academic journals, and relevant regional and international organization websites: a systematic search was conducted through Scopus, PubMed, Science Direct and Google Scholar and international sites (e.g., GCC, FAO, WHO, etc.). The literature was reviewed to identify, similar challenges, global best practices, advanced and relevant benchmarks for the Oman’s case.

Given the dynamic nature of food safety, the selection criteria for obtaining data and information focused on recently published documents that specifically address food safety regulations, official authority roles and enforcement mechanisms in Oman. Data credibility was ensured by selecting documents from recognized national and international sources and validated based on authorship, publication source, institution origin and relevance to the study case.

## 4. An Overview of the Progress of the National Food Safety Management System in the Sultanate of Oman

Any national food control system must establish clear objectives (protecting public health, safeguarding consumers, and contributing to economic development), a defined scope of work, and key components: laws and regulations, integrated laboratories, education and capacity building, as well as the presence of an effective administrative authority (or authorities) and an efficient inspection program [7]. Globally, there are three types of national administrative structures used for regulating the food sector: sector-based multi-agency systems, single-agency systems, and integrated multi-agency systems. The integrated system is considered the most effective when there is active coordination and cooperation among various entities involved in the farm-to-table chain [7].

In the Sultanate of Oman, the current situation resembles the integrated system. The national competent authority (CA) predominantly oversees all farm-to-table chain. However, it lacks the authority to coordinate inspection activities and monitor their performance in the market, which is the responsibility of the municipal sector and the Public Authority for Special Economic Zones and Free Zones. This limitation was addressed by the mandates of the National Food Safety Committee (NFSC) issued by the MD 89/2022, the main national platform for such coordination.

In the Sultanate of Oman, ensuring the safety and integrity of food products is of the government’s highest priority. In 2020, the nation experienced significant governmental restructuring aimed at streamlining the public sector framework to align with the governance objectives outlined in Oman’s Vision 2040. The vision serves as the national framework for economic and social planning spanning from 2021 to 2040, shaping national sector strategies and five-year development plans [17]. As part of this initiative, Royal Decree No. 92/2020 was issued to transfer all regulations, resources, rights, responsibilities, and assets from the Public Authority for Stores and Food Reserve, the Water Resources sector, and the Food Safety and Quality Centre (FSQC) within the Ministry of Regional Municipalities and Water Resources (MRMWR) to the newly renamed ministry, now known as the Ministry of Agriculture, Fisheries Wealth, and Water Resources (MAFWR) [11,12].

Prior to the royal decree, the enforcement of food safety legislation was overseen by a multi-agency system comprising various governmental authorities in different ministries with overlapping responsibilities and mandates [29]. This fragmented approach led to several issues, including the absence of inter-agency data sharing, an inefficient surveillance system for foodborne diseases, inadequate alignment among the different control bodies and insufficient oversight of different segments of the food supply chain. The CA ensuring food safety and quality known as the Food Safety and Quality Centre (FSQC), was established back in 2019 under Royal Decree No. 24/2019. In 2020 with the issuance of Royal Decree No. (92/2020), it was reallocated to the MAFWR with its outlined responsibilities and resources [11,12].

FSQC, since being integrated into the MAFWR, has aligned its objectives and specializations with all directorates and departments concerned with food within the Ministry. Among the most significant of these are the Agricultural Quarantine Department and the Veterinary Quarantine Department. This alignment has had a positive impact on enhancing and developing the food safety and quality system to operate in a comprehensive and integrated manner, from farm to fork across the supply chain. This is achieved through effective legislation and regulatory systems based on the “science, evidence and risk-based approach,” serving as the main framework for decision-making related to food regulation in accordance with the recommendations of the Codex Alimentarius Commission and the parent organizations (the Food and Agriculture Organization and the World Health Organization), as well as international best practices.

The center is generally responsible for establishing and enforcing various systems, controls, initiatives, and procedures that regulate and ensure the production, import, export, circulation, and consumption of safe food products of all kinds. This is achieved through preventive, regulatory, and executive measures.

The newly reformed management and enforcement system has integrated various authorities to create a comprehensive food safety framework that spans over the entire farm-to-fork continuum [12]. The integrated national food control system employs a risk-based approach to address food safety challenges across domestic, import, and potential export markets. It encompasses harmonized laws, legislations, regulations, enforcement mechanisms, partnerships with industry stakeholders and international organizations. Its governance framework supports the development and implementation of laws, regulations, standards, and monitoring systems to mitigate foodborne hazards and ensure compliance with safety and quality requirements.

### 4.1. Food Control Management

The Food Safety and Quality Centre (FSQC) administrative structure consists of five technical departments with dedicated mandates and responsibilities working collaboratively to enforce food safety and quality across all stages of food supply chain. It is responsible for executing regulatory, legislative, related standards, specifications and hygiene requirements for food businesses, food establishments and associated activities, as well as overseeing imports and exports of food commodities. The role and duties of the FSQC include implementation and monitoring policies, strategic plans and programs for food safety and quality management. Its primary mandate is the enforcement of the Food Safety Law established by Royal Decree No. 84/2008, along with its executive regulations issued under Ministerial Decision No. 2/2010. The organization is responsible for updating and developing legislation, regulations, standards, and specifications to ensure food safety and quality in line with internationally recognized criteria. The FSQC has established an effective mechanism to enforce food safety and quality policies at all stages of the food supply chain. It collaborates with relevant authorities to monitor food commodities and prevent commercial fraud throughout the production, storage, transportation, and distribution of food, and at border entry points. The FSQC conducts analytical tests on food, water and food contact materials and takes necessary action on non-compliant findings and issues licenses to compliant food establishments and transport vehicles and vessels. It engages in scientific and research studies to overcome any challenges in the field of food safety. The FSQC supervises third party laboratories that are directly affiliated with the center to validate their analytical tests and results. Permits for food-related advertising, promotional campaigns, and events are also verified and issued by the center [16].

The presence of a central laboratory with a comprehensive range of state-of-the-art analytical techniques responsible for all the analyses required to ensure compliance against the increasingly stringent quality and safety standards. The center has a dedicated department for Risk Assessment and Food Crisis Management operating on the prevention and forecasting of potential food risks in the supply chain. The Department of Food Safety Systems Development was established to qualify food establishments in ensuring their adherence to hygiene practices and the implementation of food safety management systems in their facilities. The Food Specifications and Compliance Department is also a new addition to the center. The department was established to issue and review standards, keeping up to date with food legislation and to ensure the conformity of products with standards [16].

The exceptionality of fisheries wealth in the Sultanate of Oman that lies along a coastline spanning over 3165 km at the eastern edge of the Arabian Peninsula with rich fishing grounds, has led the government to create a dedicated center with a vital role in ensuring the safety and quality of this wealth. Seafood safety and quality control measures are prioritized and in order to maintain compliance with national and international laws and regulations, a national center was established known as the Fish Quality Control Centre (FQCC) [29,30,31]. This center was established back in 1998 under the Directorate General of Fisheries Research within the Ministry of Agriculture and Fisheries Wealth (former) with regional offices in various coastal governorates [30]. It was later reallocated to the FSQC by Royal Decree No. (92/2020). The Centre plays a vital role within the FSQC in managing the seafood supply chain by enforcing quality and safety requirements on the locally produced seafood and maintaining the integrity of both imported and exported seafood products as per Ministerial Decree No. (12/2009) [29,30]. It has its own laboratory facilities that carry out all the required analysis for ensuring the integrity of the seafood products, conducts inspections, assessments, and approves seafood facilities, vessels and related activities [30]. The FQCC is designated by the FSQC to serve as the primary seafood safety regulator in the country and acts as the competent authority in the global fisheries markets. It works in collaboration with the General Directorate of Fisheries Development and the veterinarian quarantine within the General Directorate of Animal Wealth within the MAFWR in the issuance of health certificates and for monitoring [32]. The MAFWR is bounded by international agreements with the European Commission (EC), Eurasian Economic Union, the United States, and other global markets to ensure the delivery of high quality and safe products [23].

Additionally, the FSQC has set up departments distributed across all governorates of Oman with main clear mandates and responsibilities for ensuring compliance to food safety regulations [16]. They serve as the center’s key support in the various governorates in issues related to food safety and enforcement. Departments affiliated with FSQC similarly exists in all borders points (sea, air and lands) for monitoring and inspection of food safety, serving the center’s overall enforcing system [16]. The center develops and executes training, qualification programs and awareness activities and campaigns related to food safety and quality based on risk assessment for its employees and for the food industry. It collaborates with regional and international organizations to exchange information and experiences concerning the latest updates on food safety and quality [16].

**Figure 1:**
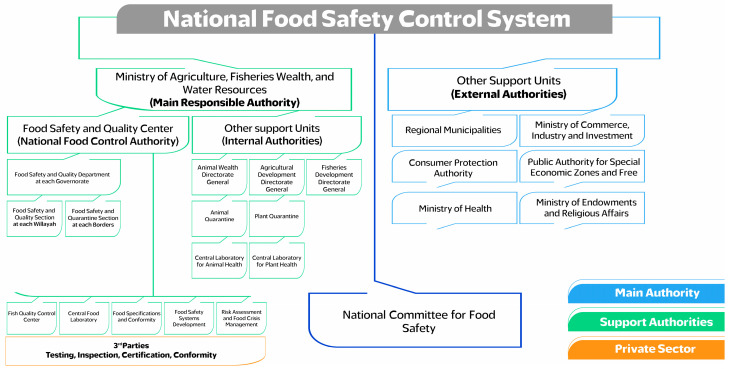
The current National Food Safety Control System (internal and external authorities).

**Figure 2:**
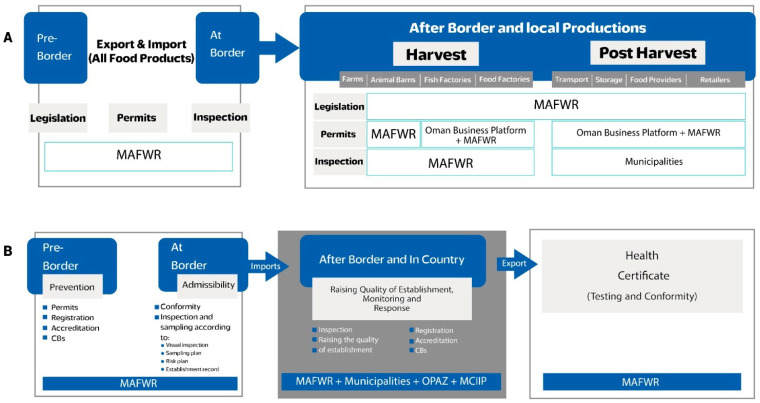
National Food Safety Management System: (**A**) roles and responsibilities and (**B**) processes across the food supply chain.

The current National Food Control System continues to share its mandates and responsibilities with various governmental authorities, including the Ministry of Commerce, Industry and Investment (MCIIP), Regional Municipalities, the Ministry of Health, the Ministry of Endowments and Regional Affairs, the Consumer Protection Authority and the Oman Public Authority for Special Economic Zones and Free Zones (OPAZ), as illustrated in Figure 1. Although the number of external authorities has decreased compared to the previous national food control structure established before Royal Decree No. 92/2020, challenges persist, and efforts are being made to address the challenges faced by the current integrated system, either through effective management and coordination with internal authorities or by collaborating with external ones [12]. Figure 2 illustrates the roles, responsibilities (A) and processes (B) carried out by the various authorities in terms of legislation, permits and inspections. The diagram depicts oversight of the Food Safety Management System throughout the food supply chain, spanning the pre-border, border, and post-border stages. The MAFWR, in collaboration with other relevant entities, holds control over most parts of the supply chain and serves as the national competent authority for food safety in the country.

### 4.2. Legislative and Policy Frameworks

The legal framework for food safety and quality in Oman consists of various laws, regulations, standards and specifications that govern various stages of the food supply chain. This framework was designed to ensure the protection of public health through promoting hygiene and best practices in food handling, production, processing, distribution and enforcement mechanisms, to enhance safety and quality across all stages of food production in the farm-to-plate continuum. It also fosters collaboration among stakeholders and international organizations [24,29].

The food safety legislation framework and the mechanism promulgated in the Sultanate of Oman in the issuance of laws, executive regulations, regulations, decisions, technical regulations and standard specifications are illustrated in Table 1. Oman has two types of legislation: primary and secondary [33]. The primary one refers to a Royal Decree (RD) and is promulgated by the Sultan, the head of state [33]. The second legislation refers to executive regulations, regulations and ministerial decisions (MD), which are promulgated by relevant ministers and government bodies in accordance with the granted authority given by the RDs, with basic laws taking precedence in a case of conflict [33,34]. Technical regulations and standard specifications are mostly promulgated by local and regional standard agencies such as the Ministry of Commerce, Industry and Investment Promotion (for local) and the Gulf Standard Organization (GSO), with the latter being a regional standard organization for the member states of the Gulf Cooperation Council and Yemen, established under the authority of the Gulf Cooperation Council [22]. It serves all member states in fostering integration and interdependence with the aim of safeguarding quality life for its member states and strengthening their economies in order to compete globally.

The issuance and implementation of food safety laws and policies are the main mandates of the FSQC. It is responsible for overseeing and enforcing these laws in coordination with relevant governmental authorities through the NFSC. In 2008, the Food Safety Law was issued by Royal Decree No. 84/2008 granting the former MRMW the authority to establish and enforce regulations ensuring consumer safety and well-being. According to Royal Decree No. 92/2020 promulgated in 2020, following the governmental restructuring, all regulations, resources, rights, and responsibilities related to food safety were transferred from the MRMWR to the MAFWR [11]. As stated earlier, the FSQC, operating under the MAFWR, serves as the national CA overseeing the entire food supply chain in Oman, from farm to fork, and ensuring compliance with national and international food safety and quality requirements as sole legislator [11]. Other authorities will adhere to this law and its executive regulations and collaborate to harmonize the legal codes across the country [12]. The NFSC works to provide legal provisions pertaining to food safety and quality by proposing appropriate measures for inspections, monitoring, and corrective actions to ensure adherence to food safety legislation and standards. The NFSC harmonizes standards by aligning technical regulations and standard specifications across the different authorities to comply with international standards. It coordinates with regional and international committees, as it is important to ensure adherence to food safety regulations and standards in the protection of consumer health and safety [29,35].

**Figure 3:**
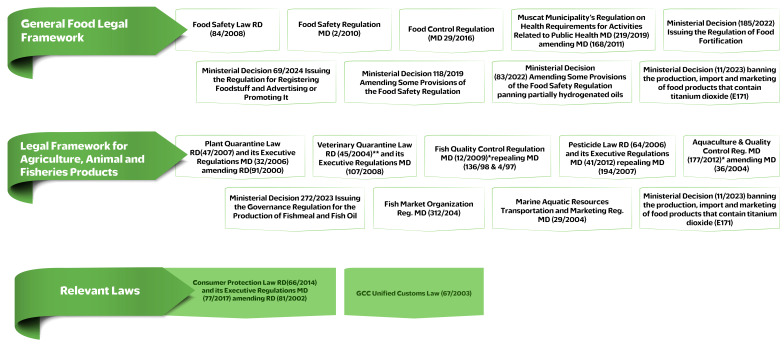
Food safety legal framework in the Sultanate of Oman. * in the process of being updated, ** in the process of being revised.

The Food Safety Law RD (84/2008), its Executive Regulations MD (2/2010) and Food Control Regulation (MD 29/2016) amending MD (241/1999) comprise general provisions and articles related to the protection of public health. They cover everything related to hygienic practices: the promotion of foodstuffs; aspects of distribution and transportation of food products and related permits; storage; compliance with standard specifications; import and export of food products; permits for the release of food shipments; inspections; withdrawal of food products from the food market and fines and penalties. Other important laws that govern the integrity and safety of food and feed were also promulgated by the MAFWR: the Veterinary Quarantine Law (RD 45/2004) and its Executive Regulations (MD 107/2008); the Plant Quarantine Law (RD 91/2000; amended in 2007 to 47/2007) and its Executive Regulations (MD 32/2006); and the Pesticide Law (RD 64/2006) and its Executive Regulations (MD 41/2012) [29]. The seafood safety division has its own regulations in place to ensure compliance with conditions and standards approved by the competent authority as shown in Figure 3. The relevant food safety authorities have also issued laws that have provisions and articles related to the protection of public well-being and are illustrated in Figure 3.

The Minister of MAFWR has issued various MDs with the latest one being MD 69/2024. This decision introduces regulations for the registration and promotion of foodstuffs, with an aim to establish a comprehensive database for tracking food products as part of a comprehensive food traceability system. The decision will streamline local, import and export processors, regulate production, enhance food safety standards and trade practices, and outline procedures for the registration and advertising of food items domestically and internationally. In addition, it will affirm the conformity of products. Together, these policy and legal frameworks create a comprehensive approach to managing food safety, ensuring that Oman’s food supply is safe, reliable, and of high quality.

However, limitations do exist in terms of outdated legislation and fragmented sector-specific food policies, thus failing to follow a holistic approach to address challenges faced by the food control systems. Candela and Pereira [36] have reviewed global challenges in food policy and suggested a holistic and integrated approach towards addressing interconnected issues such as public health, food security, sustainability and economic stability. The authors highlighted the need to adopt long-term strategies to align with global frameworks such the UN’s Sustainable Development Goals.

### 4.3. Food Monitoring and Surveillance

The monitoring and surveillance of the national food control systems aim at unifying efforts across the country and at the various border entry points, and include official inspectors, quarantine departments, and governmental and private laboratories, all under a cohesive framework. The MAFWR aims to unify all the enforcement efforts of the various authorities in and with those of the FSQC to carry a holistic enforcement mechanism through inspections, monitoring and laboratory analyses throughout the supply chain [16]. This consolidation effort is designed to ensure the implementation of essential preventive measures, mitigation of potential risks, and to enhance community health and safety. The Ministry seeks collaboration with various governmental authorities and private sectors to achieve an integrated, harmonized and effective food safety system in the country.

### 4.4. Inspection and Official Enforcement

Food inspection is crucial within national food control activities, ensuring that all the stakeholders within the food production and supply chain adhere to proper processes, gathering evidence, and verifying compliance with established standards and requirements. A risk-based inspection is founded on scientific methods and prioritizes inspection efforts on food products and establishments presenting the highest risks to consumer health. The frequency of risk-based inspections should be aligned with the food safety risks associated with food processing and the inherent risks within the process facilities with an aim of determining inspection frequency according to the evaluated risk levels for both food and facilities [5].

The Food and Quarantine Departments (FQDs), which are strategically positioned across the country, featuring maritime ports, land and air entry points, along with primary quarantine facilities located in most governorates, serve the inspection of all food, agriculture and animal consignments ensuring their safety and their being free from pests and diseases [16]. Enforcement mechanisms, including inspections, monitoring, and assessments, are currently implemented in Oman with the coordination of various authorities responsible for food safety enforcement. The integrity and safety of food commodities are maintained from the source before being granted entry to the country. Joint inspection with the relevant food safety authorities (internal and external authorities) is carried out at the borders as shown in Figure 2. It is worth noting that the FSQC is currently working on the implementation of Conformity Bodies (CBs) inside and outside the country.

Joint efforts with strategic partners, including regional municipalities responsible for food monitoring in their respective governorates, including the Consumer Protection Authority, which plays a crucial role in preventing commercial fraud in food and other sectors, and the Public Authority for Special Economic Zones and Free Zones in the industrial sector, are all coordinated under the NFSC (MD No. 45/2003) [16]. This committee unifies these enforcement authorities to fulfill their role and mandates as specified in the Food Safety Law.

#### 4.4.1. Veterinary Quarantine

FQDs are the first defensive line in the country with critical roles in ensuring food safety, prevention of the entry and spread of infectious diseases and the transmission of zoonotic diseases (transmitted from animals to humans) that impact both human and animal health. As a fundamental part of the public health strategy, veterinary quarantine acts as a protective barrier against transboundary epidemic and infectious disease in livestock and their products by the execution of food security policies, food safety, and biosecurity measures [16]. The veterinary quarantine department oversees all sea, land and air entry points, with quarantine facilities serving all the governances of the Sultanate operating under the Veterinary Quarantine Law issued by Royal Decree No. 45/2004 and its executive regulations issued by Ministerial Decision No. 107/2008 [16]. The Veterinary Quarantine Law is a unified legislation for the Gulf Cooperation Council (GCC) countries and in accordance with the recommendations of the World Organization for Animal Health (WOAH) and the Codex Alimentarius [16,29]. The VQ also serves as the Sultanate’s representative in the WOAH.

In addition to FQDs, a central animal health laboratory with veterinary quarantine capabilities has been established to enhance disease diagnosis. This initiative aims to protect public health by conducting regular examinations of locally, imported and exported animals and food from animal origin, ensuring their safety and quality while preventing the transmission of diseases to humans.

#### 4.4.2. Plant Quarantine

FQDs also function at border entry points to ensure the safety of imported, exported and transit agricultural consignments. Plant quarantine is governed under Royal Decree No. 47/2004 and its executive regulations are issued under Ministerial Decision No. 32/2006 [16]. This law provides a unified framework for the GCC countries and complies with the International Plant Protection Convention (IPPC), with the Plant Quarantine Department acting as the representative for Oman. The law aims to prevent agricultural epidemics and diseases, protect the environment and plant resources, and regulate the importation and exportation of plants and their products.

The rapid advancement of transportation and international trade have led to a significant increase in the movement of agricultural products across borders, resulting in the spread of diseases and infested plants. In response, the ministry established a central laboratory of phytosanitary and plant health to address these challenges which is operated by a private entity [16].

#### 4.4.3. Official Food Control Laboratories

Official food control laboratories are integral to the functioning of national food safety and control systems. They contribute to safeguarding public health, prevention of foodborne illnesses, regulatory compliance, and the overall safety and quality of the food supply chain. Various governmental and private laboratories serve the national food control authority in maintaining the integrity of the food products and ensuring consumer confidence. The FSQC, as the national food control authority in the country, operates internationally accredited laboratories conforming to ISO/IEC 17025 [37] standards for the analysis of official samples of food and water commodities. In the Sultanate of Oman, laboratory accreditation is overseen by the Gulf Accreditation Center (GAC), which operates in accordance with ISO/IEC 17011 [38]. It serves as the national accreditation body for the Gulf Cooperation Council (GCC) member states by providing accreditation services, such as ensuring that testing laboratories maintain technical competence and that their operational performance is aligned with global benchmarks. Most of the regulatory laboratories have adequate facilities and are well equipped with the latest instruments and devices [16,29,39], as follows:

Central food laboratory within the FSQC: Analyzes microbiological, chemical, pesticide residue, mycotoxins and radioactivity measurement for food, water and environmental samples. They respond to concerns relating to any local or imported food products that do not conform to standard specifications. Products that fail to comply with safety criteria are rejected and banned from entering local markets.

FQCC laboratories within the FSQC: Conducts rigorous analyses and monitoring of seafood products to ensure they meet national and international safety requirements. The laboratories receive samples from wild and aquaculture fishery products and carry out a residue monitoring program on local, imported and exported products. They ensure the implementation of effective biosecurity measures in the aquaculture sector to safeguard fish health, ensuring food safety and quality and ultimately contributing to the sustainability of the fisheries sector.

Quarantine laboratories: as mentioned previously, the two laboratories that function under the veterinary and plant quarantine’s work in collaboration with the FSQC in ensuring the safety of consignments entering the country at border points for plants, animals and their products.

Regional municipality laboratories: In addition to the FSQC central laboratory, ten regional laboratories allocated to various governorates under the jurisdiction of the municipalities at the governorates function in coordination with the central laboratory. They conduct analysis on food and water samples to ensure compliance with required standards and regulations.

Private laboratories: Currently, there are 21 third-party food testing laboratories operating independently from government authorities and with no affiliations to suppliers or manufacturers. They play a crucial role in the harmonizing efforts of the food control systems in Oman. Their independent status ensures credibility of the tests and analyses conducted to verify compliance with regulatory standards and specifications. A total of 16 out of the 21 private laboratories are accredited to ISO/IEC 17025 standards and are distributed across various governorates with some positioned in logistics stations.

Accreditation and inter-laboratory programs: All laboratories affiliated with the National Food Control Authority, whether governmental or private, must be accredited to international standards such as ISO/IEC 17025, as stipulated by MD 59/2011. This decision establishes regulations for the registration of examination and calibration laboratories. Any private laboratories providing testing and calibration services must be registered in accordance with this decision, under the oversight of the Directorate General of Standards and Metrology in the Ministry of Commerce, Industry, and Investment. The recognized laboratories must have formal recognition and competency to perform tests and analyses fulfilling requirements for quality and competence in producing reliable and accurate results with calibrated instruments and devices.

The affiliated laboratories are engaged in regular internal audits and assessment by FSQC officials to verify compliance with the requirements of the management system and legislation laid down the competent authority. Proficiency tests (PTs), a type of inter-laboratory comparison (ILC), are carried out periodically by the Central Food Laboratory within the FSQC with all the recognized laboratories to assess the comparability and reliability of test results which is vital for regulatory compliance and enhancing the overall integrity of the food control systems. The overall performance indicator of PT in 2023 was 98.7%, indicating an excellent performance.

#### 4.4.4. Accreditation and Qualification of Food Facilities at Source (Domestic and International)

The Fishery Quality Control Center, Food Safety Systems Development Department within the FSQC, Veterinary and Plant Quarantines, plays a critical role in ensuring adherence to health and food safety standards at the pre-border control [16]. Proactively verifying health standards and food safety at point of origin is essential before products enter the country. Effective border control in the country of origin is crucial for maintaining the integrity of the food products by enforcing regulations and standards from the source. In this regard, the FSQC has developed an initiative to raise the standards of 300 national food establishments annually.

The growing global distribution of food products necessitates inspection and testing of imported goods at both national and international borders [40]. Challenges that are usually not present in local produce may arise when dealing with imports from other countries. The strengthening of post-border or in-country controls serves as a robust defense against the transmission of infectious diseases, contaminates, unapproved veterinary pharmaceuticals in animal-derived foodstuffs or excessive levels of pesticides in plant products [40].

Domestic control, on the other hand, ensures the adoption and implementation of recognized food safety management systems throughout the entire food supply chain. This includes all primary producers, such as farms and fish farms, as well as fishing vessels, both artisanal and commercial [41]. The scope extends to processors, storage facilities, distributors and retailers. Additionally, transportation services must also adhere to specific guidelines to maintain food safety during transit. Food preparation and catering services, in coordination with regional municipalities, are also included to ensure safe food handling practices are observed in meals served to the public.

Regular inspections, training programs, and compliance audits are fundamental components of this control framework. By implementing these measures, authorities can effectively mitigate risks and safeguard public health throughout the entire farm-to-table continuum.

#### 4.4.5. Logistics Stations or Hubs

The Logistics Stations in Oman are hubs established as part of partnership between the MAFWR and the private sector. Five hubs have been built across the country with more on the way to serve the logistics sector, the regulator and the food industry [41]. The idea of establishing models for logistics cities seeks to create integrated facilities for inspection, visual and laboratory testing, as well as to provide handling, storage and sorting services according to the best global safety and quality standards [35,42]. This initiative responds to regional and global developments and aligns with key national priorities emphasizing the redefinition of the roles and relationships between the public and private sectors with the aim of supporting the ministry’s mission of fostering public–private partnerships [35,42]. Therefore, encouraging effective contributions from the private sector to enhance food and water security while maximizing economic benefits.

The hubs serve as the first line of defense for food imports and exports from various countries through ports and land crossings. Representatives from food control authorities responsible for registration, inspection of safety and quality, animal and plant health, governmental and private laboratories operate under one roof to facilitate and streamline procedures and enhance time and inspection efficiency [42].

#### 4.4.6. Traceability

Traceability as defined by the Codex Alimentarius Commission (2006) [43], is “the ability to follow the movement of a food through specified stage(s) of production, processing and distribution”. Omani legislation and food control enforcement have made significant strides in enhancing traceability, which is vital for ensuring food safety, sharing information, addressing potential risks, fostering consumer trust, and aligning with both national and global standards. An initiative known as the Food Management and Tracking Program is currently in development and will be launched soon [44]. Ministerial Decision No. 69/2024 has been issued to establish a comprehensive database for tracking food products as part of a broader food traceability system. Additionally, the establishment of logistic hubs and modern facilities reflects government efforts to implement traceability across various stages of the supply chain [35,41].

Despite these advancements, challenges remain in current traceability systems, including limited implementation in certain segments, fragmentation, and a lack of technological integration, particularly among smallholders. In contrast, modern and large food processors have adopted traceability primarily to meet market demand rather than regulatory compliance, aligning with the findings of Pham et al. in Vietnam [8].

A study was conducted in the Oman’s seafood sector to evaluate the level of implementation of seafood traceability systems in seafood establishments and the enforcement role of government officials [45]. The findings, based on qualitative questionnaires, indicated that government officials are generally effective in the enforcement of the traceability regulations. However, while seafood establishments recognize the potential benefits of the system, they face challenges in implementation, particularly in the upstream stages of the supply chain [45].

### 4.5. Management of Food Safety Emergencies and Crises

The management of food safety emergencies requires a well-structured approach to ensure public health and safety. An essential component of the Food Safety Emergency Response (FSER) as defined by FAO and WHO [46], involves assessing risks, making informed management decisions and effectively communicating risks. Emergencies and crises can be minor or major depending on the levels of impact; therefore, it is crucial that both governments and food business operators have the capabilities to prevent and control food safety risks at their early stages [46]. When food safety risks are compromised, they can expand rapidly from being a domestic issue to becoming an international incident affecting the reputation of the country in the global market.

The Risk Assessment and Food Crisis Management Department at the FSQC is one of the most important departments in addressing emerging issues and crises based on risk and science. It operates on both local and international levels by monitoring domestic and global markets, while coordinating its internal operations with relevant ministries and authorities [16].

Continuous surveillance and monitoring of food products within the supply chain aids in the early detection of potential hazards, risks and foodborne illness. Therefore, the Rapid Alert System for Food and Feed (GCC-RASFF) system for the Gulf Cooperation Council (GCC) facilitates communication and the exchange of information among member states, including Oman, Bahrain, Kuwait, Qatar, Saudi Arabia, and the UAE on issues related to food safety risks [29,32]. The platform provides data on food safety incidents, alerts on contaminated or unsafe products and allowing the transmission of information to its members, thereby encouraging collaboration among the GCC national food safety authorities and their relevant stakeholders to react promptly and effectively to ensure a unified response during emergencies. Effective communication channels among governmental officials, stakeholders and consumers are essential in saving lives. The development of an early warning system during extreme weather disasters and pandemics, enhances resilience and the availability of safe food [46], particularly as Oman has experienced extraordinary weather conditions in recent years.

### 4.6. Public Awareness and Research Development

Public awareness and advancing research in food safety are vital in the advancement of the national food control authorities. Technological advancements in food science and food safety are progressing rapidly, resulting in the adoption of new techniques, methods and technologies [1]. If these innovations are integrated and embraced by the relevant authorities and stakeholders, they will enhance their working knowledge and experience [47]. Fostering a strong culture in food safety through awareness programs, education and training can safeguard public health, improve food security and reduce food waste.

The national food control authority in Oman, in collaboration with all other concerned governmental authorities, conducts an annual Food Safety Week that includes public awareness campaigns, training workshops, and scientific conferences, the first of which took place in 2010 [29,48]. This initiative aims to raise awareness and enhance knowledge within the community about food safety and quality, public health protection, best practices, and effective responses during food safety emergencies. Additionally, it focuses on building capacity and developing resources to improve food safety efforts [48]. Similarly, other specialized conferences and seminars are held throughout the year by the competent authorities and relevant authorities.

Research development in food safety is fundamental for enhancing public health by mitigating risks associated with foodborne illnesses, addressing emerging food safety challenges and ensuring food quality [49,50]. It supports the development of evidence-based regulations and standards, ensuring compliance of food products with the safety requirements and facilitating updates to the regulatory framework. Collaboration between government authorities, academic institutions and private sector aid in research leads to the development of new technologies, and advances monitoring methods, testing, innovative food products and improving food safety practices.

The Food Safety and Quality Centre (FSQC) performs various scientific research work in collaboration with academic institutes and relevant authorities [35]. A key initiative in this effort is the Strategic Research Project Program, which is supported and funded by the Ministry of Higher Education, Research, and Innovation. The program aligns with Oman Vision 2040 with the aim of addressing strategic priorities across multiple sectors, including health, food and water security, energy security, water research, and artificial intelligence technology. The program’s outcomes are expected to greatly enhance evidence-based decision-making and improve efficiency and productivity by providing innovative solutions to government challenges [51]. Additionally, FSQC research studies receive funding from the Agricultural and Fisheries Development Fund, an independent agency that plays a crucial role in promoting innovation, development, and sustainability within the agriculture, livestock, fisheries sectors, and food security [52].

### 4.7. Achievements, Challenges and Future Directions of the National Food Control System

#### 4.7.1. Achievements

Oman’s national food safety control system has witnessed substantial progress in enhancing the safety and quality of food products from the farm-to-table continuum. It has evolved significantly over the past few years, strengthening its food safety control systems through a combination of effective legislation (laws, regulations, technical regulation, and standards), infrastructure development, advanced technology, capacity building and international cooperation. In recognition of the deficiencies in the previous institutional framework governing the food safety system, Royal Decree No. 92/2020 was issued to address the establishment of an integrated food control system, overcoming the previous multi-agency system [12]. Similarly, several countries have adopted similar approaches to unify their national food control systems, shifting towards a more risk-based approach, as seen in the work of [8,9,53].

The Sultanate of Oman has been an active member in the Codex since 1972. It took on the role of Coordinator of the FAO/WHO Codex Coordinating Committee for the Near East during the 47th session of the Codex Alimentarius Commission that was held in Geneva, Switzerland, 25–30 November 2024. This shows Oman’s journey with Codex and the commitment to regional and global collaboration, supporting food safety and quality and enabling consumer protection and fair practices in the food trade.

The period post-2020 has marked a significant transformation in Oman’s national food control system. The transformation has been driven by the country’s broader goals to fulfill Oman Vision 2040, and the needs of addressing emerging of food safety risks, improving public health and enhancing food security. Al Busaidi and Jukes [29], reported that prior to 2020, the food control system in Oman was characterized as a fragmented entity with overlapping responsibilities spread across various governmental authorities. These authorities often had roles, with the laws and regulations related to food safety and quality issued by various governmental bodies. Enforcement of these regulations, on the other hand, was carried out by different authorities in the form of a multi-agency system. The system was comprised of the Ministry of Regional Municipalities and Water Resources (MRMWR) as the primary authority for food safety, along with the Ministry of Agriculture and Fisheries Wealth (MAFW), the Ministry of Commerce and Industry (MOCAI), the Ministry of Health (MOH), local municipalities (Muscat, Sohar and Dhofar), the Public Authority for Consumer Protection (PACP), and the Royal Oman Police (ROP) [29]. This is a situation faced by many countries around the globe, prompting numerous efforts to unify and establish a unified or integrated system [8,9].

Digitalization and real time monitoring systems are highly effective tools in monitoring food safety, enabling the tracking of non-compliant food businesses, processors, foodborne illnesses and contamination risks. The national food control center is on the verge of implementing these systems to enhance its response to emerging health risks related to food [44]. One such initiative is the Food Management and Tracking Program.

Post-2020, monitoring surveillance has been enhanced with a focus on a risk-based inspection system and more digitized real-time monitoring schemes. Rapid response systems provide better communication among regulatory agencies to enable more responsive and effective handling of a food safety crisis. More achievements are highlighted in Table 2, with differences that were made prior to 2020 and the changes that occurred following the issuance of Royal Decree No. 92/2020. The table illustrates the transformation before and after the decree, comparing key differences and improvements.

#### 4.7.2. Challenges

Despite the achievements in food safety governance, the national food control system encounters several challenges that need to be addressed. Effective enforcement and compliance with food safety legislation is one of the primary challenges, regardless of the existence of comprehensive regulations in the country. The ineffectiveness could be due to resource constraints, limited capacity, and the complexity of the food supply and distribution chain, monitoring the abuse of the cold chain, and the issues of the cold chain in a warm climate. Moreover, capacity-building, logistical constraints and inadequate training may hinder the ability of the enforcement authorities to carry out inspections, monitoring compliance and enforcing penalties. The absence of a risk assessment center that can provide scientific-based opinion on emerging food issues is a limiting factor toward a resilient and efficient control system. Legislative gaps, overlaps and outdated regulations may present challenges besides the lack of coordination in food safety mandates among the relevant authorities. Challenges related to supply chain management, emerging threats and technological advancements are some of the several challenges that are still faced by the national food safety control authority. For instance, weak monitoring of certain segments of the food supply chain due to resource constraints may enhance food fraud and adulteration, thus introducing new hazards and risks that require stronger regulatory controls and preventive measures. The lack of an effective traceability system in certain segments of the supply chain within the country may hinder the ability to track the origin of food items and their movement throughout the supply chain, causing delays in addressing safety concerns. The need for advanced and continuously evolving food safety measures in all their aspects is of paramount importance in addressing the new risks with the increasing complexity of the global food supply chain and rapid technological advancements, with more challenges addressed in Table 2.

Similar challenges have been highlighted by Barinda and Ayuningtyas [54], emphasizing regulatory gaps, overlaps, and resource limitations that hinder effective food safety management in Indonesia’s food control system. Hai and Dinh [8] provide an analysis of the food control system in Vietnam, forecasting achievements and remaining issues such as weak enforcement, insufficient resources, fragmented regulations, and a lack of public awareness that continue to undermine the effectiveness of the food control system. Alrobaish et al. [53] examined the food safety governance system in Saudi Arabia, with a particular focus on the challenges associated with controlling imported food. They identified key challenges as being inadequate regulatory frameworks, limited enforcement capacity, insufficient coordination between authorities, and gaps in monitoring and inspection systems for imported food products. Given the similar challenges faced by various countries, a more effective regulatory framework with an enhanced internal food safety control system, and greater collaboration among relevant stakeholders, may enhance risk mitigation and safeguard food safety.

## 5. Discussion

The evolution of the food safety management system in the Sultanate of Oman from a fragmented, multi-agency system to an integrated food control system conforms to the global trend recommended by FAO and WHO [1,3,6,7]. Various studies highlight the limitations of multi-agency systems, particularly in terms of overlapping mandates, insufficient use of resources, and inconsistent enforcement practices [8,29,36,39,54].

In Oman, this transition was formalized through Royal Decree No. 92/2020 leading to substantial improvements in the overall structure. The improvements included a streamlined integrated approach with well-defined roles and responsibilities, alignment with national policies, and international standards with a risk-based approach. This shift has reportedly demonstrated significant developments in the coordination, efficiency and effective food safety measures across the entire food supply chain, including domestically produced, imported and exported products [12]. These changes, in turn, have strengthened crisis response mechanisms and enabled more effective risk-based assessment [16], similar to outcomes observed in other countries that adopted an integrated and more unified food safety control system [8,27,28].

Although the integrated system has brought various improvements in the national food control system, limitations in certain aspects remain. Synthesized findings from various studies and academic literature point to a gap in technical capacity building, particularly in specialized technical expertise, insufficient resources, and a lack of integrated data management systems as being key limitations, among others [29,36,39,54].

Table 2 provides a comparative analysis of the current food safety management system in Oman, contrasting the pre-2020 system with the post-2020 transformation, and mapped against international benchmarks [1,3,23,47]. It highlights the key components of an effective food safety system in terms of food control management, legislative and policy framework, inspection and official enforcement, official food control laboratories, public awareness, and research development [7]. The table illustrates the evolution from a less coordinated multi-agency model to a more unified, risk-based system that meets international expectations and criteria. While foundation improvements have been achieved, some constraints still exist. Therefore, a need to invest in capacity-building, infrastructure, data and risk communication is crucial to sustain the gains and improvements [1,6].

## 6. Conclusions

Oman’s transition from a multi-agency food safety system to an integrated food control system represents a significant step toward ensuring the health and safety of its population. The integrated approach, led by Royal Decree No. 92/2020 restructuring the food safety management system in the country, has improved coordination, overcoming fragmented regulations, data sharing, and the overall effectiveness of food safety measures. Aligning food safety with plant and animal quarantines has established better coordination of food safety systems. In addition, the country’s legislation and policies are increasingly harmonized with regional and international organizations such as those led by the Gulf Cooperation Council (GCC) and Codex Alimentarius to standardize and monitor food safety practices, ensuring consistency with global best practices, and facilitate cross-border cooperation.

To support these structural reforms, Oman has developed and constructed essential infrastructure, including logistics hubs, a national food safety database, and both governmental and private ISO accredited laboratory facilities. However, to fully recognize the potential of these advancements, targeted investments are needed in areas such as artificial intelligence, real-time monitoring and surveillance systems, sufficient resources, training and public engagement. Technologies like artificial sensors, such as e-nose, e-tongue, and vision systems, offer promising tools for rapid assessment of food quality; however, their implementation requires feasible assessment of their cost, technical capacity and regulatory approval. Additionally, addressing emerging new food sources, such as insects, lab-grown meat, and precision fermented foods, requires careful consideration of safety and cultural acceptability.

## 7. Future Directions

Building on the progress achieved through structural reforms and the integration of the food control system, Oman’s future efforts should focus on strengthening technological innovation, enhancing institutional capacity and international collaboration. The development of artificial intelligence (AI) capabilities in the Sultanate of Oman will strengthen and enhance the long-term sustainability of its integrated food safety and food security systems. AI applications may include risk prediction, supply chain monitoring and food security assessment. Effective implementation requires strategic collaborations among technology providers, regulatory authorities, academic institutions and food industry stakeholders across the food supply chain [55]. The development of a centralized big data platform encompassing information across the food supply chain on food production, stability, safety and associated risks is essential. With such a platform, AI tools can support real-time data analysis, interpretation, response, infrastructure control measures, monitor and integrate information through technology, devices and human inputs [56]. These capabilities can enhance monitoring and risk assessment skills across the whole food supply chain. Oman has initiated activities in this direction by launching the first version of the Food Safety Hackathon in 2024 as part of its “Food Safety Week” with an aim to encourage innovation and developing digital solutions in addressing food safety challenges [57].

The development of infrastructure facilities and resources is important in enabling rapid monitoring and response. Using advanced analytical tools, such as artificial sensors, (e-nose, e-tongue), computer vision systems and natural language processing technologies may support and enhance accuracy in food safety assessment [56].

Globally, the emergence of alternative food sources such as edible insects, lab grown or cell culture meat, cheese, milk, mycoproteins, and precision fermentation, are gaining attention as future foods and are part of addressing food security and environmental sustainability [58]. This technology could use any sort of bioresources or waste to convert them into different types of food ingredients [59]. Regulatory frameworks should have clear strategies to address safety, nutritional, biological and chemical risk factors associated with these emerging products [58].

In this context, technological and regulatory collaboration and partnership at the regional and international levels is essential. The Sultanate of Oman has signed bilateral agreements with various countries aimed at enhancing food security and safety. These agreements, particularly within the Gulf Cooperation Council (GCC) may facilitate the exchange of knowledge, technology, food safety standard harmonization and capacity development in alignment with international best practices.

## Figures and Tables

**Table 1:** Food safety legislation framework in Oman (Source [12,33]).

Omani Food Safety Legislation	Description	Authorities Responsible of Issuance
**Law**	The legal foundation for any system or rules that regulate the conduct of a community, outlining mandates and responsibilities of enforcement and the powers of regulatory bodies, as well as penalties for non-compliance.	Royal Decree (RD) promulgated by the Sultan
**Executive Regulation**	Rules or directives issued by a government or regulatory authority to implement and enforce laws passed by the legislative body.	Ministerial Decision (MD) by Relevant Ministries
**Regulation**	Refers to a rule or directive created and enforced by a governmental authority to control or govern conduct within its jurisdiction. Includes specific regulations providing detailed requirements for food safety practices, including hygiene standards, labeling requirements, permissible limits for contaminants, etc.	
**Decision**	A formal conclusion or resolution made by an authority or governing body on a specific issue or case.	
**Technical Regulation**	Refers to a set of specific requirements, standards, procedures, product characteristics or their related processes and production methods and applicable administrative provisions, established by a regulatory authority, for which compliance is mandatory, to ensure safety, quality, and performance in various sectors, including food safety, manufacturing, and consumer products.	Ministry of Commerce, Industry and Investment Promotion and Gulf Standard Organization (GSO)
**Standard Specification**	National standards for food quality and safety are aligned with international benchmarks to ensure consistency and reliability. These standards cover aspects such as food additives, packaging materials, and nutritional information.	

**Table 2:** Comparative analysis of food safety management systems: Pre-2020 vs. Post-2020 transformation based on international guidelines [1,3,23,47].

Components of Analyses	Pre-2020 (Multi-Agency)	Post-2020 (Integrated Food Control System)
**Food Control Management**		
Administrative structure	• Multiple agencies • Fragmented, with overlapping responsibilities • Limited defined roles and responsibilities of the different authorities involved in the food control management • Establishment of food safety committee by the MRMWR with representatives from the various authorities with outlined mandates and responsibilities	• Streamlined, integrated approach • Aligning with international standards • Well-defined roles and responsibilities of the different authorities involved in food control management
Allocation of resources in terms of financial, human, equipment, information, etc.	• Adequate • Limited qualified human resources	• Adequate • Limited qualified human resources • Decentralization leading to properly utilized resources
Scientific principles and risk analysis approach	• Lacks this approach • Lacks the appropriate infrastructure for risk assessment data	• Responsive with integrated systems and inter-agency collaboration • A dedicated department for risk assessment and food crisis management • Legal provisions are structured on scientific bases
Food safety crisis response	• Slow, less coordinated response	• Responsive with integrated systems and inter-agency collaboration • A dedicated department for risk assessment and food crisis management operating on the prevention and forecasting of potential food risks in the supply chain
Integrated food chain approach covering the entire farm to plate continuum	• Difficult to assess due to the scattering of the food control system among the different authorities and within, hence creating many gaps on enforcement within the food chain	• Better integration due to one authority in charge with clear inter-agency collaboration; however, a certain degree of overlapping and gaps still exists
Involvement of the various stakeholders from farm to plate continuum in decision making process and flow of information	• Limited	• Enhanced involvement due to the allocation of food safety authority with animal and plant quarantines within the same ministry
Active involvement in regional and international standard-setting bodies on issues related to food safety and quality	• Very active regionally and internationally • An active member of the GCC, GSO, WTO (TBT & SPS), WHO, FAO, ROMPE, IAEA, CAC, WOAH, and IPPC *	• Similarly • An active leading role in Codex Alimentarius plus the involvement of private sector.
Surveillance of food-borne illnesses (microbial, chemical, allergen and etc.) from primary production to consumption	• Food-borne illness surveillance is mainly carried by the Ministry of Health with no collaboration with other authorities	• Improved coordination and follow-up with Ministry of Health through active committees and communication platforms
Existence of a national database that consolidates all data generated from enforcement and laboratory activities.	• Does not exist on a national level • Mostly available at different authorities’ level	• Available for food control activities carried out by the FSQC • Available also at the different authorities’ levels involved in the food control management
**2.** **Legislative and Policy Frameworks**		
Ensure a high level of health protection and safeguard consumer interests	• Adequate	• Improved (e.g., Food Fortification program and banning of titanium dioxide)
The roles and responsibilities of government authorities responsible for food control within the food safety control management systems along with the mechanisms and procedures for their interactions	• The roles and responsibilities of the government authorities are not clearly defined. • There is no established mechanism for interaction; however, a food safety committee has been created as a common platform to bring together the focal points from different authorities.	• Similarly • The law has not been updated since its issuance in 2008 to reflect the changes introduced by Royal Decree No. 92/2020.
Existence of an integrated and comprehensive legislation that covers the entire farm-to-table continuum	• Legislative gaps and overlaps • Lack of integration of the existing legislation, as each authority within the food safety control management systems enacts its own regulations. • Food Safety (84/2008), however, is the legal legislation that serves as the principal legal framework for enforcing food safety control measures in the country	• Existence of integrated legal frameworks creating a comprehensive approach to managing food safety, ensuring safe, reliable, and high quality food supply • A unified food law has been adopted by the Ministerial Committee for Food Safety of the Gulf Cooperation Council (GCC) member states as a mandatory basis with an aim of enhancing enforcement and monitoring practices within the member states
Technical regulations and standards are based on sound science and comply with Codex Alimentarius Commission (CAC)	• Harmonized its technical regulations and standards in line with CAC through the GSO. • Incorporated various technical regulations and standards from the EU directives and laws pertaining to seafood safety and quality	• Similarly
Sanctions and penalties enforcement	• Addressed; however, it is considered too lenient and insufficient to effectively encourage compliance with the related legislation	• Similarly
Outlines clear provisions on the responsibility for food safety and quality lies with producers and processors	• Adequate	• Adequate
Provides clear provision for the approval, registration or licensing of food premises	• Adequate	• Adequate
Provides clear provisions on traceability and recall procedures in case of safety issues	• Absent	• Present but limited • An initiative will be launched soon “Food Management and Tracking Program,” for tracing and tracking food • Ministerial Decision No. 69/2024 has been issued to establish a comprehensive database for tracking food products as part of a broader food traceability system
Includes obligations ensuring that only safe and fairly presented foods are placed in the market	• Clearly outlined but limited in implementation	• Limited • Ministerial Decision No. 69/2024 has been issued to establish a comprehensive database for tracking food products as part of a broader food traceability system
Recognized country’s international obligations particularly to trade	• Adequate	• Adequate
Legislation in line with international standard	• Adequate	• Adequate
Contains provisions for detailed enforcement procedures	• Present but limited	• Present but limited
**3.** **Inspection and official enforcement**		
Inspection based on risk analysis including sampling programs and techniques for domestically-produced, imported and exported food	• Not based on risk analysis • Majority of the inspectors have limited knowledge of modern risk-based approach	• An entire department was introduced for risk assessment. • Risk assessment training program was introduced. • Risk-based sampling protocol implemented.
Roles and responsibilities of the inspection activities are clearly defined	• Activities and roles are not well defined and overlap with those of other relative authorities • Inspectors operating independently, with overlapping responsibilities and some are assigned multiple tasks, rather than being dedicated exclusively to inspecting food premises and processors.	• Inspectors have dedicated roles and responsibilities within the FSQC and in collaboration with their relevant counterparts. • Clear SOPs are set between the two main inspection authorities (FSQC and Municipalities)
Inspection activities encompass the entire farm-to-table approach	• Fragmented through a number of governmental agencies, therefore creating gaps and not fulfilling the farm-to-table approach • Insufficient human resources to cover the whole chain due to lack of organizing the existence resources	• Even with one authority in charge, gaps exist due to lack of cooperation within the ministry and with other authorities of OFCS. However, clear SOPs are set between the two main inspection authorities (FSQC and Municipalities) • Insufficient resources to cover the whole chain
Requirement for qualified and trained inspectors	• Most inspectors have basic qualifications; however, there are higher qualified inspectors although limited in numbers	• Similarly • Training program well defined
Reputation and integrity of the inspectors	• Adequate	• Adequate
Number of official inspectors authorized to carry out the enforcement duties within the food safety control systems	• Not sufficient	• Not sufficient • Involvement of third parties (CBs) will be implemented
Existence of Inspection Standard Operating Procedures (SOPs)/manuals	• Absence	• Absence, however, clear SOPs are set between the two main inspection authorities (FSQC and Municipalities)
A comprehensive understanding of existing food laws and regulations	• Adequate	• Adequate
Development of digitalization and real-time monitoring	• Paper-based inspection • Limited use of digital tools, basic inspection systems	• An attempt to implement advanced automation, and real-time data monitoring systems (Food Management and Tracking Program)
Presence of a national database that categorizes food premises based on the risk level of the produced food products	• Exists within each authority	• Exists within the authority
Access to logistics for conducting inspections including resources, facilities, transportation modes, inspection equipment and devices	• Mostly available but limited in certain circumstances	• Adapting the use of logistic stations
Existence of records and documentation covering various aspects of inspection activities, such as consumer complaints, investigation and management of outbreaks of food-borne illnesses, respond to and manage food emergencies, etc.	• Limited, particularly the records and documents on the management of food-borne illnesses outbreaks and food crisis and emergencies	• Similarly • Adapting Food Management and Tracking Program
Presence of a review and evaluation mechanism for the food inspection system	• Absence	• Absence
**4.** **Official food control laboratories**		
Adequate number and strategic placement of official food control laboratories to support the food control system	• Limited	• Adequate
Presence of reference laboratories for contaminants and food-borne disease causative agents	• Presence with limitation	• Presence of a reference laboratory at Ministry of Health for food-borne illness outbreak investigation
Accreditation of official food control laboratories according to international standards	• Most laboratories are in the process of obtaining ISO 17025; however, none of them have been accredited	• All laboratories, including third-party labs, are accredited with ISO 17025, while analyses are not accepted unless the laboratories are accredited
Qualified food analysts with appropriate training, experience and integrity	• Adequate	• Adequate
Adequate infrastructure, facilities, equipment, supplies, reference materials, and participation in inter-laboratory proficiency testing	• Mostly available	• Adequate
Access to calibration and maintenance services for equipment and instruments	• Calibration and maintenance of the instrumentation is carried out by the instrument providers and lack international recognition • Calibration of some of the instruments and devices is carried out by Directorate General for Specifications and Measurements (DGSM), within the Ministry of Commerce and Industry and Investment Promotion	• Similarly
Analytical methods for analysis of various contaminants are validated	• Most analyses in the different laboratories use validated and reference methods	• Validated and reference methods
Presence of standard operating procedures (SOPs) for all analytical methods	• All the official laboratories have SOPs in place for all the analytical methods and instrumentation used	• Similarly
Effective coordination and collaboration between official food control laboratories and the enforcement officials	• Each official laboratory falls under a specific authority and has effective linkage with the administrators and food inspectors within that authorities • However, weak linkage and collaboration among the various official laboratories	• Effective coordination and collaboration between FSQC Central Laboratory and private laboratories
Effective coordination and collaboration between official food control laboratories and the public health system for food-borne disease surveillance, as well as any other relevant laboratories	• No effective linkage • Article 21 of the Food Safety Law (84/2008) emphasized the need to coordinate among the various official laboratories and submitting the analytical results to the food safety committee for further actions	• FSQC Central Laboratory plays a major role in coordinating between private laboratories and the Ministry of Health
**5.** **Public Awareness and Research Development**		
Presence of extension and developing programs for implementing information, education, and communication (IEC) activities	• Each authority within the official food safety control system has its own extension programs to conduct IEC for various stakeholders • Seminars, workshops and conferences are also organized to educate the FSC staff and others on relevant food safety issues • A food safety week is carried out annually to educate and raise awareness about food safety, targeting various stakeholders within the food industry and relevant sectors. • Oman Association for Consumer Protection (OACP) is an independent, voluntarily and non-government organization that protects the consumers’ rights and raises awareness on various issues, including food safety	• Similarly • FSQC initiated a holistic capacity building program
There is a policy in place for IEC regarding food safety and quality, targeting external audiences such as consumers, NGOs, the food industry, and others	• No policy exists but the NGOs are carrying out awareness campaigns for educational purposes	• Similarly • Higher level of awareness campaign is witnessed
Availability of sufficient financial resources, appropriate materials and equipment to carry out IEC activities	• Limited	• Adequate
Sufficiently trained FSC staff to carry out IEC	• Limited	• Adequate
A risk communication system in place to manage food crises and emergencies	• Limited	• An entire department was introduced for risk assessment • Risk assessment training program was introduced • Risk-based sampling protocol in implemented
Presence of dedicated research institutions or departments focused on food safety and quality	• Present but limited	• A new dedicated department for research was established at FSQC
Investment in food safety research	• Limited	• Presence of funding agencies such the Agricultural and Fisheries Development Fund and the Strategic Research Project Program Funds, which supported all studies conducted in the field of food safety and quality
Collaboration with academic and research institutions	• Present but limited	• Collaboration with local and international institutions
Innovation in food safety practices	• Absence	• Present but limited

* GCC: Gulf Cooperation Council, GSO: Gulf Standardization Organization, WTO: World Trade Organization, TBT: Technical Barriers to Trade Agreement, SPS: Sanitary and Phytosanitary Measures Agreement, ROMPE: Regional Organization for the Protection of the Marine Environment, WHO: World Health Organization, IAEA: International Atomic Energy Agency, CAC: Codex Alimentarius Commission, WOAH: World Organization for Animal Health, IPPC: International Plant Protection Convention, FAO: Food and Agriculture Organization.

## Data Availability

No new data were created or analyzed in this study. Data sharing is not applicable to this article.

## Abbreviations

The following abbreviations are used in this manuscript:

GCC	Gulf Cooperation Council
CAC	Codex Alimentarius Commission
WOAH	World Organization for Animal Health
GSO	Gulf Standardization Organization
WTO	World Trade Organization
TBT	Technical Barriers to Trade Agreement
SPS	Sanitary and Phytosanitary Measures Agreement
ROMPE	Regional Organization for the Protection of the Marine Environment
EU	European Union
IAEA	International Atomic Energy Agency
IPPC	International Plant Protection Convention
WHO	World Health Organization
FAO	Food and Agriculture Organization
MAFWR	Ministry of Agriculture, Fisheries and Water Resources
GRASFF	Rapid Alert System for Food and Feed
NFSC	National Food Safety Committee
FSQC	Food Safety and Quality Centre
MRMWR	Ministry of Regional Municipalities and Water Resources
MCIIP	Ministry of Commerce, Industry and Investment Promotion
OPAZ	Oman Public Authority for Special Economic Zones and Free Zones
FQDs	Food and Quarantine Departments
MOH	Ministry of Health
PACP	Public Authority for Consumer Protection
ROP	Royal Oman Police
